# Experimental modelling studies on the removal of dyes and heavy metal ions using ZnFe_2_O_4_ nanoparticles

**DOI:** 10.1038/s41598-022-10036-y

**Published:** 2022-04-09

**Authors:** Xiaoyu Zhao, Leila Baharinikoo, Meysam Davoodabadi Farahani, Bentolhoda Mahdizadeh, Amir Abbas Kazemzadeh Farizhandi

**Affiliations:** 1 Department of Food and Pharmaceutical Engineering, Suihua University, Suihua, 152061 Heilongjiang China; 2 Department of Analytical Chemistry, Faculty of Chemistry, University of Mazandaran, Babolsar, Iran; 3 Faculty of Mining, Petroleum and Geophysics Engineering, Shahrood University of Technology, Shahrood, Iran; 4 Department of Biomedical Engineering, South Tehran Branch, Islamic Azad University, Tehran, Iran; 5 Computer Science Department, Boise State University, 777 W Main St, Boise, ID 83702 USA

**Keywords:** Environmental sciences, Chemistry

## Abstract

The presence of dyes and heavy metals in water sources as pollutants is harmful to human and animal health. Therefore, this study aimed to evaluate the efficacy of zinc ferrite (ZnFe_2_O_4_) nanoparticles (ZF-NPs) due to their outstanding properties including cost-effectiveness, availability, and applicability for removal of auramine O (AO), methylene blue (MB), and Cd (II). The effect of the main operating parameters such as AO concentration, MB concentration, Cd (II) concentration, adsorbent amount, solution pH, and sonication time was optimized by the response surface methodology (RSM). Optimal conditions were obtained at adsorbent amount of 0.25 g, pH = 6, sonication time of 15 min, and concentration of 15 mg L^−1^, and more than 91.56% were removed from all three analytes. The adsorption of AO, MB, and Cd (II) onto ZF-NPs followed pseudo-second-order kinetics and the equilibrium data fitted well with Langmuir isotherm. The maximum adsorption capacities of ZF-NPs for AO, MB and Cd (II) were as high as 201.29 mg g^−1^, 256.76 mg g^−1^ and 152.48 mg g^−1^, respectively. Also, the reuse of the adsorbent was investigated, and it was found that the adsorbent can be used for up to five cycles. Based on the results of interference studies, it was found that different ions do not have a significant effect on the removal of AO, MB, and Cd (II) in optimal conditions. The ZF-NPs was investigated successfully to remove AO, MB, and Cd (II) from environmental water samples. The results of this study showed that ZF-NPs can be used as a suitable adsorbent to remove AO, MB, and Cd (II) from aqueous solution.

## Introduction

Environmental pollution created by the disposal of dyes and heavy metals has become one of the major global concerns, threatening the ecosystem organisms and human health^1,2^. Over the past few decades, the growing use of dyes and heavy metals has led to the increase of these compounds in groundwaters and surface waters, which caused severe health and ecological hazards. It is necessary to prevent the release of these toxic substances into the aquatic environment and separate them from the wastewaters due to the increased water demand and limited water resources^3^.

Heavy metals are stable elements that tend to accumulate in biological organisms. Heavy metals like cadmium are also toxic in trace amounts. Heavy metals poisoning can seriously damage the central nervous system, lungs, kidneys, and other vital organs. Moreover, the release of these contaminants into water sources will threaten the health of aquatics^4^. Dyes are one of the major pollutants present in the wastewater of some industries like paper, plastic, leather, food, and textile industries that cause significant pollution of the aquatic environment^5^.

Annually, about 700,000 tonnes of dyes of more than 10,000 types are produced globally, and about 1–15% of these dyes are released into the wastewaters during the dyeing process. Auramine O (AO) and methylene blue (MB) are the most widely used dyes in the industry. Respiratory irritation, teratogenicity, and carcinogenicity in humans are the main drawbacks of poisoning by these compounds^6,7^. Moreover, the presence of these dyes in water sources would lead to blocking the light penetration into the water, disruption of photosynthesis, and destruction of the aquatic ecosystem. For these reasons, it is essential to apply appropriate methods to remove contaminants such as dyes and heavy metals^8^.

In order to remove heavy metals and dyes from aqueous solutions, there are various methods such as adsorption, ion exchange, reverse osmosis, coagulation and flocculation, advanced oxidation, and ultra-filtration^9–13^. Considering cost-effectiveness, the adsorption method is the simple, safe, ideal, and economical option for removing dyes and metal ions from contaminated water^14^.

In the last decade, iron oxides (Fe_2_O_3_) have drawn a lot of attention due to their catalytic properties to address environmental issues^15^. Ferrites are magnetic compounds whose main constituent is iron oxide. Easy separation by an external magnetic field after reaction and recovery is one of the most essential advantages of these compounds in water and wastewater treatments. Zinc ferrite (ZnFe_2_O_4_) is widely used in water treatment processes due to its non-toxicity, high phase resistance, visible light absorption, low cost, insolubility in water, and light corrosion resistance^16,17^.

Wang and Shih (2021) used ZnFe_2_O_4_/rGO Nanocomposites to adsorb methylene blue (MB) dye. The results revealed that the efficiency of the adsorption process increases with reaction time, pH, and the amount of adsorbent; however, increasing the initial concentration of dye significantly decreases the efficiency of adsorption. In optimum conditions of pH = 8, initial concentration = 10 mg L^−1^, adsorbent dose = 10 mg, reaction time = 30 min and temperatur = 298 K, the removal efficiency of 98% was achieved. The results showed that ZnFe_2_O_4_/rGO Nanocomposites could act as a readily available, eco-friendly, and cost-effective adsorbent to remove dyes from wastewater of various industries^18^.

In another study, Habibi et al. (2021) applied ZnFe_2_O_4_ to remove Congo Red (CR) dye from the aqueous medium. In this study, the effect of different parameters like pH, initial dye concentration, and contact time on the removal efficiency was evaluated. The results showed that in optimum conditions of pH = 3.5, adsorbent dose = 0.006 g, dye concentration = 60 mg L^−1^, and contact time = 90 min, the highest dye removal efficiency was obtained^19^.

Jethave et al. (2019) applied Pb@ZnFe_2_O_4_ nanocomposites for the removal of Congo Red (CR) dye. The central composite design was applied to evaluate and optimize the effect of various factors such as pH, adsorbent mass, dye concentration, and contact time. The optimized parameters (dye concentration = 150 mg L^−1^, pH = 7.1, agitation time = 90 min, and adsorbent mass = 50 mg) results in 96.49% removal of the dye from the solution. The results showed that Pb@ZnFe_2_O_4_ nanocomposites could be utilized as a useful adsorbent in treating aqueous samples^20^.

Konicki et al. (2017) evaluated the application of ZnFe_2_O_4_ nanocomposite in removing Rhodamine B (RB) from wastewater. The effect of individual parameters such as dye concentration, reaction temperature, and pH were investigated. The results displayed that the adsorption behaviour of the RB onto ZnFe_2_O_4_ nanocomposite fitted well with the Langmuir isotherm model, with the maximum adsorption capacity of 12.1 mg g^−1^. The obtained results showed that ZnFe_2_O_4_ nanocomposite could be used as a highly efficient adsorbent for industrial wastewater treatment^21^.

In recent years, the removal of pollutants from the aquatic environment by ultrasonic waves (ultrasound-assisted) is a new technique that has attracted a lot of attention^22,23^. Ultrasound is the sound above the range of human hearing (20 kHz–100 MHz). Cost-effectiveness, increased removal speed, decreased removal time, and increased removal efficiency are the main benefits of ultrasonic waves^24^. Due to the valuable applications of ultrasound technology in wastewater treatment, in this study, pollutants were removed by ZF-NPS in the presence of ultrasound waves.

The design of experiments (DOE) improves the system and process performance to ensure optimal results. In traditional methods of evaluating the effect of parameters on the process, the influence of factors on each other is not considered. In conventional methods, only one factor is changed, and the other parameters are kept constant. Moreover, the colossal number of experiments are time and cost-consuming and require a lot of materials. To solve these problems, the multivariate statistical technique is highly beneficial, the best of which is the response surface methodology (RSM), which is based on a set of statistical and mathematical methods^25–27^. In this method, the optimal value for each variable is achieved by selecting the effective variables in the process and conducting the experiment based on the design matrix.

In this study, the efficiency of ZF-NPs in removing AO, MB, and Cd (II) with the help of ultrasonic waves was investigated. RSM was used to model and assess the effect of operational parameters. Effective parameters such as pH, ZF-NPs amount, analyte concentration, and ultrasonic time were considered as the independent variables, and the removal percentage (% R) was considered as the response. The efficiency of this adsorbent in removing AO, MB, and Cd (II) in the presence of interfering ions in environmental water samples. RSM was used to create a function between variables and response. Furthermore, the optimum conditions of this process were determined.

## Materials and methods

### Materials and instruments

In this study, all the reagents include methylene blue dye (≥ 82%), auramine O dye (≥ 80%), cadmium nitrate tetrahydrate (99%), zinc acetate (≥ 99%), iron (III) chloride hexahydrate (≥ 98%), hydrochloric acid (37%) and sodium hydroxide (≥ 97%) was obtained from Merck Company (Darmstadt, Germany). To conduct the experiments, the stock solution of 1000 mg L^−1^ was prepared individually from dyes and heavy metals; then the working solutions were prepared by diluting the stock solution. HCl (0.1 M) and NaOH (0.1 M) solutions were used to adjust the pH, and it was measured by a pH meter. In order to measure the concentration of dyes and metal ions in the experimental stages, UV–Vis spectrophotometer and atomic absorption spectroscopy (AAS) was used, respectively. An ultrasonic device equipped with a digital timer was used to disperse the adsorbent in the solvent. Also, a centrifuge was used to accelerate the settling of the adsorbent. The characterization of ZF-NPS was performed using a scanning electron microscope (SEM), X-ray diffraction (XRD), Fourier transform infrared (FT-IR) spectroscopy, Energy-dispersive X-ray analysis (EDX), and Brunauer–Emmett–Teller (BET) techniques.

### Synthesis of ZnFe_2_O_4_ nanoparticles (ZF-NPS)

Zinc ferrite nanocrystals were prepared by the hydrothermal method. For this purpose, 25 mL iron (III) chloride hexahydrate (0.4 M) was mixed with 25 mL zinc acetate (0.2 M). Then, 25 mL sodium hydroxide solution (3 M) was added dropwise to the above mixture. A red precipitate was formed and placed in Teflon autoclave at 300 °C for 12 h. In the end, the final precipitate was washed several times with distilled water and dried in an oven at 80 °C. The synthesized ZnFe_2_O_4_ nanoparticles were characterized by SEM, XRD, FT-IR, BET, XPS, and EDX.

### Removal experiments

#### Batch experiments

In this study, experiments were done individually for each analyte. In order to perform the adsorption process, an Erlenmeyer flask (250 mL) charged with 100 mL of dye or heavy metal solution with given concentrations based on the design of experiments performed by RSM was used. pH adjustment in the range of 2 to 10 was performed with HCl (0.1 M) and NaOH (0.1 M) solutions. In each experiment, a given amount of ZF-NPS was transferred to the Erlenmeyer flask, and sampling was done at the specified time. In all experiments, an ultrasonic batch was used to disperse the adsorbent in the solvent. After the specified time, the ZF-NPS magnetic sorbent was quickly removed by an external magnetic field. In each experiment, a specific volume of samples was taken to continue the process and centrifuged at 5,000 rpm for 5 min. Finally, the concentrations of dyes and metal ions in the remaining solution were measured by UV–Vis spectrophotometer and atomic absorption spectroscopy (AAS) techniques, respectively. The removal percentage of analyte from the samples was calculated using Eq. 1.

1 $$ \% {\text{R}} = \frac{{{\text{c}}_{{\text{o}}} - {\text{c}}_{{\text{e}}} }}{{{\text{c}}_{{\text{o}}} }} \times 100 $$

Also, the amount of pollutant adsorbed per gram of the ZF-NPS was calculated based on Eq. 2.

2 $$ {\text{Qe }} = \frac{{\left( {C_{0} - C_{e} } \right)V}}{M} $$

where, C_0_ (mg L^−1^) is the analyte concentration in the initial solution, C_e_ (mg L^−1^) is the equilibrium concentration of analyte in the solution at different time, V (L) is the volume of the solution, and M (g) is the weight of ZF-NPS.

#### Response surface methodology (RSM) based on central composite design (CCD)

The relationship between a response and the value of one or more factors is called response surface. The response surface is usually represented by three dimensions function and is used to fit the experimental data. In this method, the level or range of variables are determined, and defining the best range will lead to achieving a response close to the optimal point. At this stage, after selecting the level and approaching near the optimal point, the researcher needs a model that provides a high-precision approximation of the actual behaviour. Given that the existing response surface has a curvature near the optimum point, simple linear models are not adequate to study this process. In these cases, a quadratic model for data fitting is usually appropriate for approximating the response (Eq. 3).

3 $$ Y \, = + \mathop \sum \limits_{i = 1}^{k} \beta_{i} X_{i} + \mathop \sum \limits_{i = 1}^{k} \beta_{ii} X_{i}^{2} + \mathop \sum \limits_{i \le j}^{k} \mathop \sum \limits_{j}^{k} \beta ijX_{i} X_{j} + e $$

In this equation, *y* is the predicted response of each factor (removal percentage), *i* is a linear coefficient, *j* is a quadratic coefficient, *β*_*0*_ is regression coefficient, *β*_*i*_ is linear effect, *β*_*ii*_ is quadratic effect, *β*_*ij*_ is linear interaction, *x*_*i*_ and *x*_*j*_ are encoded values of independent variables, *k* is the number of investigated and optimized factors in the experiment, and *e* is the residual error. Central composite design (CCD) is often used to create second-order polynomials. In this design, each variable is tested at five levels, and there are three different types of points, including factorial points, central points and axial points. Factorial points are at the vertices of the coordinate system axes, and are coded as − 1 and + 1. The central point (0) is at the center of the design space. Axial points at a distance α from the center of the design cube are on the axes of the coordinate system, and these points are used to calculate the curvature.

## Results and discussion

### Characterization of the ZnFe_2_O_4_ nanoparticles (ZF-NPS)

**Figure 1:**
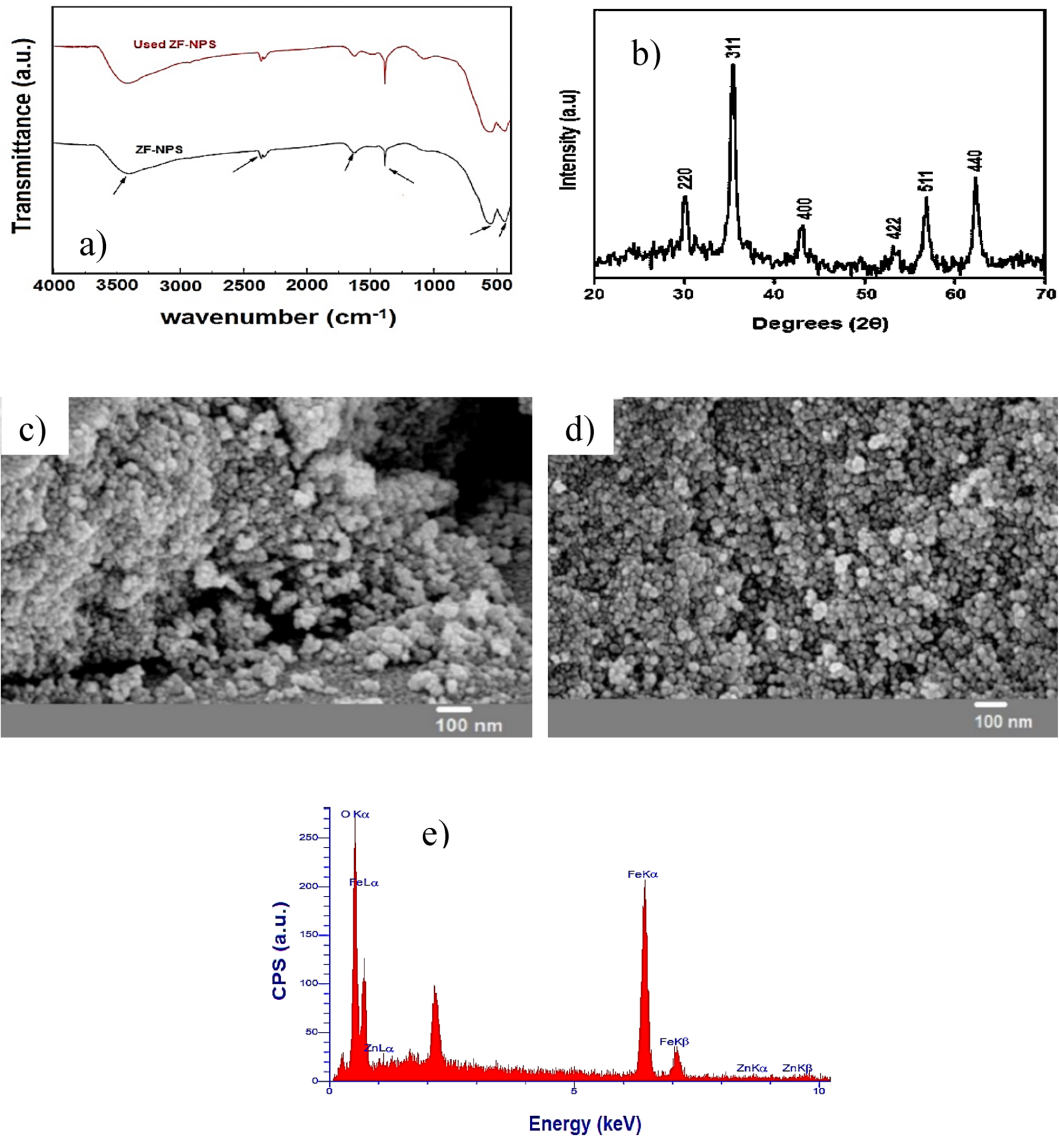
(**a**) FT-IR spectra, (**b**) X-ray diffraction pattern, (**c**), The SEM image of fresh ZF-NPS, (**d**) The SEM image of used ZF-NPS, and e) EDX analysis.

Fourier transform infrared (FT-IR) spectroscopy offers useful information about chemical groups, including high-polarity bands or bands such as the C=O and O–H groups whose dipolar moment change during vibration. Figure 1a shows the FT-IR spectrum of ZF-NPS and used ZF-NPS. As shown in the spectrum, the absorption broad band at 3420 cm^−1^ is attributed to the stretching vibrations of H_2_O molecules and OH groups of the ZF-NPS surface. The band at ~ 1600 cm^−1^ corresponds to the bending vibrations of H_2_O molecules. The band at ~ 2360 cm^−1^ indicates the CO_2_ adsorption from the air and the band at 1380 cm^−1^ indicates the presence of nitrate. The band at about 436 cm^−1^ can be attributed to the stretching vibrations of octahedral Fe^3+^ (Fe–O mode). Moreover, the observed band at about 564 cm^−1^ can be attributed to the stretching vibrations of tetrahedral Zn^2+^ (Zn–O mode). Furthermore, there is no difference in the peaks of the FTIR curves before and after the process. This result showed that ZF-NPS has excellent chemical stability and could be used as a reusable adsorbent in most applications.

The sample structure was examined by X-ray diffraction (XRD) analysis. XRD patterns were recorded in the range of 2θ = 20° to 70°. X-ray diffraction pattern of ZF-NPS (Fig. 1b) revealed that diffraction peaks at 2θ values of 30.1°, 35.3°, 43.0°, 53.3°, 56.8°, and 62.8° could be indexed with (220), (311), (400), (422), (511), and (440) planes of the cubic spinel ZnFe_2_O_4_, respectively. X-ray diffraction pattern of ZF-NPS matched well with the standard card (JCPDS: 01-077-0011). According to the ZF-NPS X-ray diffraction pattern, no impurity peak was observed, so the expected structure was formed successfully. Moreover, the approximate size of the ZF-NPS was 17.23 nm according to the Debay–Scherer equation.

Morphological analysis of ZF-NPS was performed by scanning electron microscopy (SEM) analysis. Figure 1c,d shows the SEM images of the ZF-NPS and used ZF-NPS. In these images, irregular texture, several pores, and crystallinity of the sample are observed. In addition, a comparison of SEM images of ZF-NPS and used ZF-NPS showed that there was no significant difference between the fresh and used adsorbent, which indicates that ZF-NPS is very stable during the adsorption process.

Figure 1e shows the energy-dispersive X-ray (EDX) spectrum of the ZF-NPS. The EDX spectrum identifies the elements present in the composition. According to Fig. 1e, iron, oxygen, and zinc are the only elements in the product. According to EDX results, the percentage of each element is Fe (46.37%), O (52.18%), and Zn (1.45%). As expected, the percentage of zinc is very low, and the presence of Fe and O is visible. These results indicate the Fe_2_O_3_ synthesis.

To examine the chemical composition of the surface and the elemental valence of the adsorbent, X-ray photoelectron spectroscopy (XPS) analysis is performed. Therefore, the elemental composition and the chemical state of ZF-NPS before and after the reaction were investigated by XPS analysis. Figure S1a Survey XPS spectrum of ZF-NPS confirms the predominant presence of Zn, Fe, and O elements. In Fig. S1b, the line shape of the O 1 s core surface resembled a Gaussian with a binding energy of 530.4 eV, indicating the presence of oxygen at ZF-NPS. Figure S1c shows two distinct peaks at 1022.5 eV and 1045.4 eV which is attributed to the Zn 2p_3/2_ and Zn 2p_1/2_ spectra, respectively. As can be seen, there is no significant change in the binding energies of the O 1 s and Zn 2p peaks before and after the reaction. The peaks at 725.6 eV and 719.0 eV are attributed to Fe 2p_1/2_ and the shake-up satellite structure of ZF-NPS, respectively (Fig. S1d). For Fe 2p_3/2_ of ZF-NPS before reaction, four peaks at 713.6 eV, 712.3 eV, 711.3 eV, and 710.3 eV were fitted for the spectrum. The peak at 710.3 eV is attributed to the presence of Fe (II) oxide and the other three peaks are attributed to the Fe (III) oxide.

The structural information of ZF-NPS was accessed by N_2_ gas adsorption at 77.3 K in the relative pressure range (P/P_0_) of 0.998. The test sample was degassed at 120 °C for two hours. Specific surface area, total pore volume, and mean pore diameter were measured by the standard BET method. Based on this analysis, the surface area, total pore volume, and mean pore diameter of the sample were 85.46 m^2^ g^−1^, 0.1426 cm^3^ g^−1^, and 23.8 nm, respectively.

### Central composite design (CCD) and analysis of variance (ANOVA)

Experimental design through the CCD method to investigate the adsorbent efficiency in the removal of contaminants was accommodated with Design-Expert software (Version 10.0.6, State-Ease, Inc., USA). The levels of four independent variables including, sonication time, pH, adsorbent dosage, analyte concentration, and results, are given in Table 1. In this study, the total number of experiments was found to be 30 runs. To increase the accuracy and validity of the test results, each experiment was repeated three times. The results of experimental runs and the results of the software prediction for each analyte are given in Table 1S.

**Table 1:** The design of CCD.

Variables		Unit		Range and levels				
				− α	− 1	0	+ 1	+ α
A- Adsorbent amount		g		0.10	0.15	0.20	0.25	0.30
B- Analyte concentration		mg L^−1^		10	20	30	40	50
C- pH of the solution		–		2	4	6	8	10
D- Sonication time		min		10	25	40	55	70

Run	Space type	A (g)	B (mg L^−1^)	C	D (min)	%R_AO_	%R_MB_	%R_Cd (II)_
1	Center	0	0	0	0	84.36	95.09	82.61
2	Factorial	1	1	− 1	1	54.76	50.47	55.64
3	Axial	− 2	0	0	0	33.05	25.23	43.18
4	Axial	2	0	0	0	74.13	67.68	80.41
5	Factorial	1	1	− 1	− 1	36.41	33.19	39.36
6	Factorial	− 1	1	1	− 1	33.95	39.50	37.57
7	Factorial	− 1	1	− 1	− 1	18.37	19.94	25.61
8	Center	0	0	0	0	82.23	92.42	82.05
9	Center	0	0	0	0	81.70	93.11	84.39
10	Axial	0	0	2	0	61.45	65.41	66.29
11	Factorial	− 1	− 1	− 1	1	52.09	48.75	52.50
12	Axial	0	0	0	− 2	45.37	55.76	48.07
13	Factorial	1	1	1	− 1	53.86	61.31	56.40
14	Factorial	− 1	1	1	1	52.25	47.69	49.61
15	Factorial	− 1	− 1	1	1	69.57	74.08	69.59
16	Factorial	1	− 1	1	1	93.61	95.20	90.17
17	Factorial	− 1	− 1	− 1	− 1	34.37	33.45	43.58
18	Factorial	1	− 1	− 1	1	73.53	71.09	72.72
19	Factorial	− 1	− 1	1	− 1	50.72	58.38	60.87
20	Center	0	0	0	0	82.46	91.42	81.16
21	Axial	0	2	0	0	46.92	43.85	47.23
22	Center	0	0	0	0	81.03	92.28	83.59
23	Axial	0	0	0	2	78.34	85.14	73.86
24	Center	0	0	0	0	83.11	91.89	81.97
25	Factorial	1	− 1	1	− 1	74.35	87.33	77.81
26	Factorial	− 1	1	− 1	1	34.83	31.71	39.48
27	Axial	0	− 2	0	0	87.81	88.42	81.91
28	Factorial	1	− 1	− 1	− 1	60.95	59.19	58.34
29	Factorial	1	1	1	1	72.42	71.65	72.50
30	Axial	0	0	− 2	0	23.63	22.98	34.49

In this study, the obtained data of the polynomial model were analyzed using analysis of variance (ANOVA) (Tables S2 and S3). F-value compares the model variance and the residual variance (error) and is calculated by dividing these two parameters. The closer the variances, the lower the F-value. Therefore, the higher F-value for a response shows that the variables and their levels have a more significant effect on the response^28,29^. As can be seen from Tables S2 and S3, the F-values for removing AO, MB, and Cd (II) were 261.05, 246.68, and 158.18, respectively. These values indicate the importance of the model, besides, the variables and their levels have a significant effect on the response. Like F-value, the *P* value also determines the effect of process variables on the response. The lower the *P* value, the greater the statistical significance of the variables. The P-value for all models is less than 0.05, which means that the model and variables are statistically significant. In general, the higher the F-value and consequently the lower the *P* value of a variable, the greater the significance of the variable^30,31^. According to the above table, the coefficient of determination (R^2^) for all three models is greater than 99.33% and is so close to one. The coefficient of determination measures the proportion of the changes described by the model relative to the mean (overall mean response). The closer R^2^ is to 1 implies the better the performance and the greater model’s compatibility with the experimental data. R^2^ greater than 99.33% means that more than 99.33% of the data variability is well described by the model, and therefore less than 0.67% of the observed variability cannot be explained by the model. It should be noted that a high R^2^ value does not necessarily show that the model is good, as R^2^ increases with the addition of variables to the model (regardless of whether the variables are important or not). On the contrary to the coefficient of determination, the Adjusted coefficient (R^2^-Adj) has more validity to examine the adequacy of the model^32^. The R^2^-Adj value for all models was greater than 98.70%. R^2^-Adj is statistical data that does not increase with the addition of variables and would decrease even if insignificant variables are added to the model. R^2^-Adj is appropriate statistical data in evaluating the effect of decreasing or increasing the model variables in more complex experiments. The R^2^-Adj value is close to R^2^. The difference between these two values indicates the presence of unimportant variables in the model. The predicted R^2^ (R^2^-Pred) for all models was greater than 96.43%, which shows that the model can define or predict more than 96.43% of the changes (differences) of new data in the experimental range. Statistically, the difference between R^2^-Pred and R^2^-Adj should be less than 0.2. As can be seen, their difference in this model is much less than 0.2 and so is acceptable. According to the results, the selected models are sufficiently accurate for AO, MB, and Cd (II) removal and provide a good prediction of the results. Also, the equations of the prediction of the AO, MB, and Cd (II) removal percentages are given in Eqs. 4–6.

4 $$ \begin{aligned} \% {\text{Removal - AO}} & = + 82.48 + 10.66*{\text{A }} - 9.75*{\text{B}} + 8.79*{\text{C}} + 8.58*{\text{D}} - 1.10*{\text{AB}} + 0.11*{\text{AC}} - 0.16*{\text{AD}} + 0.05*{\text{BC}} + 0.20*{\text{BD}} \\ & \quad + 0.61*{\text{CD}} - 7.59*{\text{A}}^{2} - 4.14*{\text{B}}^{2} - 10.35*{\text{C}}^{2} - 5.52*{\text{D}}^{2} \\ \end{aligned} $$

5 $$ \begin{aligned} \% {\text{Removal - MB}} & = + 92.70 \, + 10.86*{\text{A }} - 10.88*{\text{B }} + 11.34*{\text{C }} + 6.54*{\text{D }} - 1.27*{\text{AB }} + 0.98*{\text{AC }} - 0.22*{\text{AD }} - 1.10*{\text{BC}} \\ & \quad - 0.19*{\text{BD }} - 0.88*{\text{CD }} - 11.83*{\text{A}}^{2} - 6.91*{\text{B}}^{2} - 12.39*{\text{C}}^{2} - 5.83*{\text{D}}^{2} \\ \end{aligned} $$

6 $$ \begin{aligned} \% {\text{Removal - Cd}}\left( {{\text{II}}} \right) & = + 82.62 + 9.10*{\text{A}} - 9.11*{\text{B }} + 7.95*{\text{C }} + 6.42*{\text{D }} - 0.05*{\text{AB}} + 0.89*{\text{AC}} + 0.97*{\text{AD}} - 0.95*{\text{BC}} \\ & \quad + 0.86*{\text{BD }} - 0.26*{\text{CD }} - 5.72*{\text{A}}^{2} - 5.02*{\text{B}}^{2} - 8.57*{\text{C}}^{2} - 5.92*{\text{D}}^{2} \\ \end{aligned} $$

The next step in studying and evaluating the proposed hypotheses and model is the analysis of residuals. For statistical assumptions, it is recommended to examine the normal probability plot of residuals and the residual plots versus the predicted values^33,34^.

**Figure 2:**
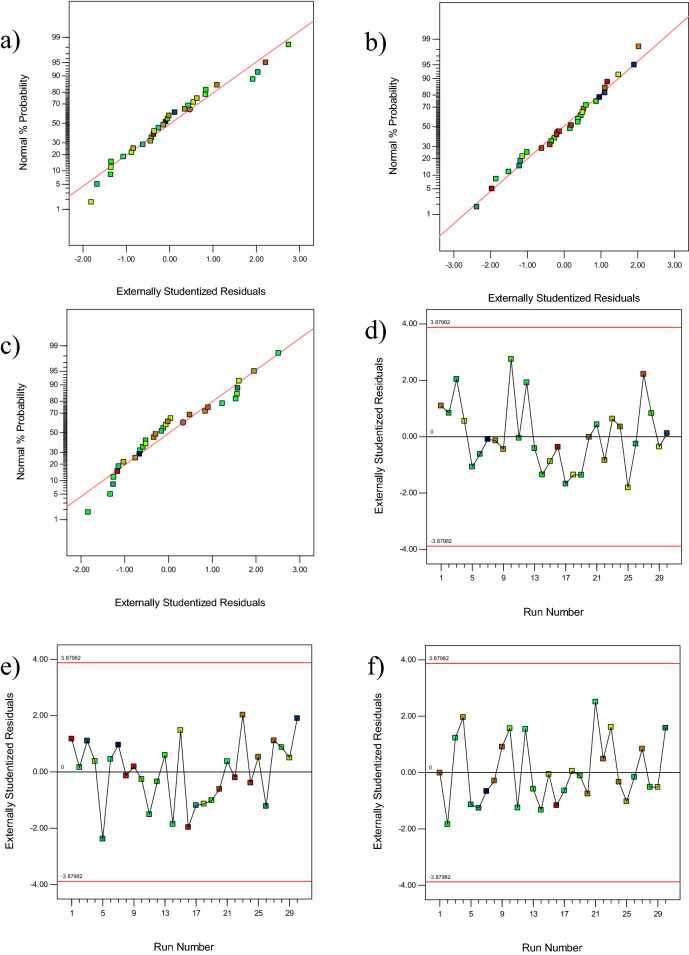
The normal plot of residuals for removal of (**a**) AO, (**b**) MB, (**c**) Cd (II), Plot of residuals versus run number for removal of (**d**) AO, (**e**) MB (**f**) Cd (II).

The residual is defined as the difference between the observed response (actual) and the predicted value by the model. Examination of residuals should be performed in any analysis of variance. In the normal probability plot of residuals, if the model is efficient and suitable, the residuals should be unstructured. That is, their distribution should be normal, and their arrangement should not follow a specific pattern. In the case of normal distribution of errors, the general shape of the plot and the arrangement of the errors would be like a straight line, and in this straight line, the main emphasis is on the center values of the graph than the other points (axial and factorial). The presence of an outlier point is an issue that should be considered in the normal probability plot, which occurs when one of the residuals is much larger than the others. Being one or more points outside the range could distort the analysis of variance. Figure 2a-c shows the normal probability plot of residuals for the removal percentage of AO, MB, and Cd (II). It can be seen that the residuals are adjacent to the straight line, and there is no deviation from the straight line. Moreover, there are no outlier points, and the plot shows a normal distribution of errors.

Moreover, plotting the residuals according to the order of the experiments is utilized to determine the correlation between the residuals. In the case of correlation between the residuals, the assumption that the errors are independent is ignored, and in this case, the accumulation of points at one end of the plot is greater than the other end. Running experiments in random is an important factor in the independence of errors and prevent this destructive effect^35,36^. As can be seen from Fig. 2d,f, the points do not follow a specific pattern and are arranged in a scattered manner. Figure S2a-c displays the actual response values versus the predicted response values. The points must be distributed uniformly along a straight line, so that the line passes through most of the points. This plot shows how the model will predict data. According to this plot, the selected model can predict the results similar to experimental data.

### Effect of selected variables on the response (removal percentage)

**Figure 3:**
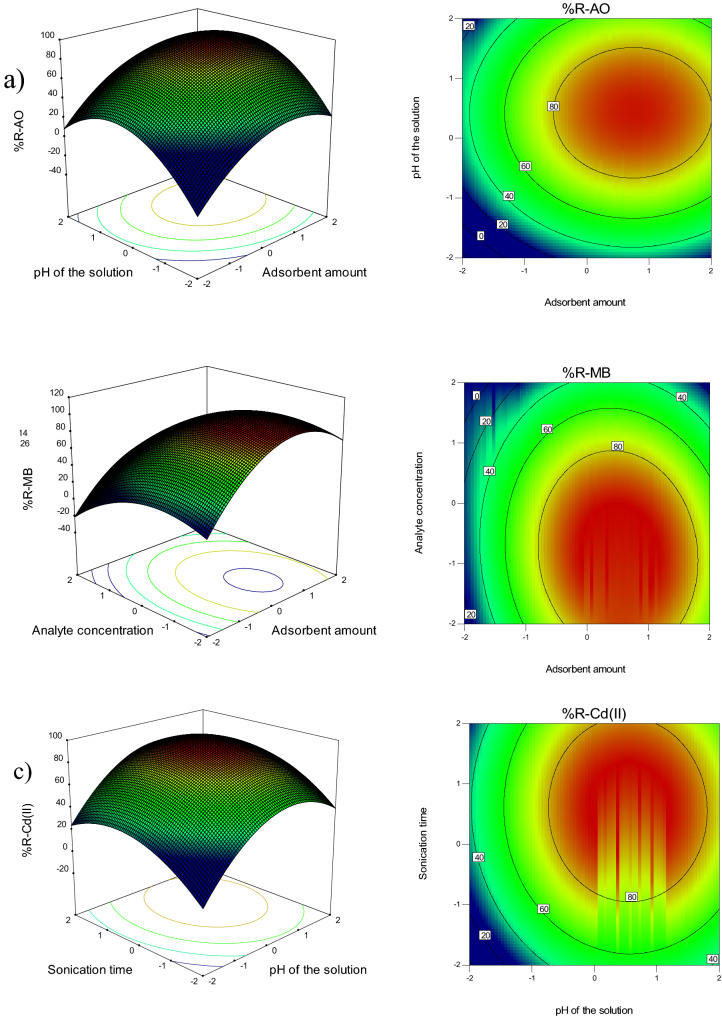
Three-dimensional plots and contour plots of the interaction effects between variables and removal (Removal conditions: adsorbent amount 0.25 g, pH = 6, sonication time 15 min, and concentration 15 mg L^−1^).

Three-dimensional plots and contour plots are drawn to better examine the design of the experiment (Fig. 3). In these plots, the effect of two factors on the response is investigated simultaneously. Figure 3a shows the simultaneous effect of the adsorbent amount and the pH of the solution on the amount of AO dye removal. One of the important parameters that have a significant effect on the adsorption process is the adsorbent dosage. The results of the effect of ZF-NPS on the removal of AO dye showed that the percentage of dye removal increases with increasing the adsorbent dosage from 0.01 to 0.03 g. As the adsorbent amount increases, the number of free surfaces to adsorb will increase until all of the dye molecules are adsorbed on the active sites of the adsorbent surface. Similar results have been observed by Khan et al. (2020), who evaluate the effect of adsorbent amount on cadmium removal. In this study, magnetic biochar modified with molybdenum disulfide was used as the adsorbent. According to the obtained results, increasing the amount of adsorbent lead to an increase in the amount of cadmium adsorption on the adsorbent^37^. In another study, Asfaram et al. (2017) utilized Mn@CuS/ZnS-NC-AC as an adsorbent to remove methylene blue (MB) and malachite green (MG) dyes. The effect of different parameters like pH, sonication time, MG concentration, Mn@CuS/ZnS-NC-AC dosage, and MB concentration on the removal rate was explored. The results showed that increasing the adsorbent dosage increased the amount of dye removal, which is consistent with the results of the present study^38^.

The effect of pH on the adsorption process of AO dye revealed that the removal percentage increased with increasing pH. The highest percentage of AO dye removal was achieved at pH 6 (Fig. 3a). The pHpzc of ZF-NPS is 5.8. So, at pH > 5.8, the adsorbent surface is negatively charged, and at pH < 5.8, the adsorbent surface is positively charged. Since AO and MB dyes are cationic molecules, the electrostatic repulsion between the adsorbent and the analyte is the main reason for increasing the removal percentage at pH > pHpzc. On the other hand, at low pHs, the electrostatic repulsion between the positively charged metal ion and the positive charge of the adsorbent surface lowered the Cd (II) removal rate. As the pH increases, the metal ion removal efficiency first increases, and then decreases, due to the formation of the insoluble cadmium hydroxide (Cd(OH)_2_) at pHs above 6. Bagheri et al. (2020) examined the adsorption of lead and cadmium from aqueous solution using rGO-FeO/Fe_3_O_4_-PEI nanocomposite, and the obtained results showed that the removal percentage of lead and cadmium decreases at alkaline pH^39^. Palma-Anaya et al. (2017), Shi et al. (2016), Fathi et al. (2015), and Rahimi et al. (2015) also obtained similar results about increasing the removal percentage by decreasing pH^40–43^.

According to Fig. 3b, the removal rate has decreased with increasing the analyte concentration. Based on these results, the adsorbent efficiency is higher at low concentrations. At lower concentrations, the available sites on the adsorbent surface are more, so the removal percentage would increase. At higher concentrations, as the number of adsorbent sites rather than the existing ions decreases and the adsorption occurs in pores deeper than the adsorbent, the adsorption process and hence the removal percentage decreases. Similar results were obtained by Khalifa et al. (2020), who used mesoporous silica nanoparticles modified with dibenzoylmethane for the removal of Cd (II), Hg (II), and Cu (II) ions^44^. Arabkhani and Asfaram (2020) applied a three-dimensional magnetic polymer aerogel as an adsorbent for MG dye removal. The effect of various parameters such as adsorbent dosage, initial dye concentration, temperature, contact time, and pH of dye solution on MG removal was examined. The results revealed that with the increase of initial dye concentration, the removal percentage decreased, which is consistent with the results of the present study^45^. Mohebali et al. (2019) used an adsorbent based on natural and cetyltrimethylammonium bromide (CTAB) modified adsorbent, celery (Apium graveolens) residue to remove congo red (CR) from the aqueous solution. The adsorbent dosage, pH, contact time, temperature, and dye concentration were investigated by adsorption experiments in a batch system. The obtained results showed that the percentage of dye removal decreases with increasing dye concentration^46^.

Figure 3c shows the effective interaction between pH and sonication time on MB removal. Sonication time is one of the main variables in the adsorption process. The effect of sonication time on the removal of metal ions and dye by ZF-NPS revealed that the rate of analyte removal increased with increasing sonication time, and it was constant at higher times. Therefore, it can be concluded that the adsorption process is performed in two stages. The fast first stage is the adsorption on the adsorbent surface, and the slow second stage is the internal mass transfer. In the first stage, most of the adsorbent sites are empty, so the adsorption process occurs fast on the adsorbent. Over time and the gradual filling of these sites, the penetration of the analyte between the adsorbed analytes and the empty sites slowed down, which led to the constant adsorption process. These results are consistent with the results of Asfaram et al. (2015), who removed auramine-O (AO) dye by ultrasound from aqueous solutions. Asfaram et al. used ZnS:Cu nanoparticles loaded on activated carbon (ZnS:Cu-NP-AC) as an adsorbent. The effective variables and the designed experiments were determined by the response surface method. Adsorbent dosage (0.02 g), ultrasound time (3 min), dye concentration (20 mg L^−1^) and pH = 7 were considered as optimum conditions. The results of this study showed that with increasing ultrasound time, the amount of AO dye removal increases. The high adsorption capacity of ZnS:Cu-NP-AC (183.15 mg g^−1^) makes it a highly potential adsorbent for the removal of AO dyes from wastewater samples^47^. In another study, Kumar et al. (2011) used activated carbon prepared from cashew nutshells as an adsorbent to remove methylene blue (MB) dye from the aqueous solution. In this study, the amount of dye removal increases with increasing contact time and the adsorbent dosage, which is consistent with the results of the present study^48^.

### Adsorption isotherms

The equilibrium adsorption isotherm is critical, as fundamental investigation explaining the interactive behavior between the absorbent and adsorption. Langmuir (7) and Freundlich (8) equations were used to analyze the isotherms data for the adsorption isotherm studies, respectively.

7 $$ \frac{{{\text{C}}_{{\text{e}}} }}{{{\text{Q}}_{{\text{e}}} }} = \frac{1}{{{\text{bQ}}_{{{\text{max}}}} }} + \frac{{{\text{C}}_{{\text{e}}} }}{{{\text{Q}}_{{{\text{max}}}} }} $$

8 $$ {\text{Ln}}\;{\text{Q}}_{{\text{e}}} = \ln \;{\text{K}}_{{\text{f}}} + \left( \frac{1}{n} \right)\;{\text{ln}}\;{\text{C}}_{{\text{e}}} $$

where *C*_*e*_ represents the metal ions’ equilibrium concentration in solution (mg L^−1^), and *Q*_*e*_ denotes the equilibrium adsorption capacity (mg g^−1^). *b* (L mg^−1^) and *Q*_*max*_ (mg g^−1^) are the Langmuir constant representing the adsorption energy and capacity. *K*_*f*_ is the Freundlich constant denoting adsorption capacity^45,49,50^. In order to investigate the parameters of adsorption isotherms, six different concentrations close to the optimum concentration were selected for each analyte, and the adsorption of these different concentrations was investigated under optimal conditions of other effective parameters. To describe the adsorption process, the desired isotherm was determined according to the correlation coefficient of the linear model of the common isotherm equations. Table 2 presents the correlation coefficients (R) and fitting model parameters of these two models. The Langmuir model’s correlation coefficients (R) for all pollutants are more than 0.98, however, they are less than 0.9 in Freundlich. In addition, the highest Langmuir model-determined adsorption values are quite closer to the capacity of all organic dyes and heavy metal ions observed experimentally. The data of adsorption are well consistent with the Langmuir model indicating that the pollutants adsorption over ZF-NPs happened as a single monolayer. In Langmuir isotherm, it is assumed that metal ions uptake happens at definite homogeneous adsorption sites via monolayer adsorption with no interaction between the absorbed molecules. Moreover, for AO, MB and Cd (II), the maximum adsorption capacity reached 201.29 mg g^−1^, 256.76 mg g^−1^ and 152.48 mg g^−1^, respectively.

**Table 2:** Parameters of isotherm model for adsorption of AO, MB, and Cd (II) on ZF-NPS.

Pollutants	Langmuir parameters			Freundlich parameters		
	Q_max_ (mg g^−1^)	B (L mg^−1^)	R^2^	K_F_ (mg g^−1^)	1/n	R^2^
AO	201.29	0.16	0.9896	33.14	0.2341	0.8324
MB	256.76	0.14	0.9884	73.01	0.2524	0.9125
Cd (II)	152.48	0.53	0.9995	30.27	0.2158	0.7631

### Adsorption kinetics

Adsorption kinetics is a key parameter for practically using the adsorbent for describing the equilibrium and rate. Applying the intraparticle diffusion kinetic, pseudo-first-order and pseudo-second-order models, the adsorption kinetics were analyzed. Moreover, the kinetic mechanism controlling the adsorption procedure is described. The intraparticle diffusion (9), pseudo-first-order (10) and pseudo-second-order (11) kinetic models are represented as:

9 $$ Q_{t} = k_{i} t^{1/2} + C $$

10 $$ {\text{log}}\;\left( {{\text{Q}}_{{\text{e}}} - {\text{ Q}}_{{\text{t}}} } \right) = {\text{ log }} - \frac{{{\text{k}}_{1} {\text{t}}}}{2.303} $$

11 $$ \frac{{\text{t}}}{{{\text{Q}}_{{\text{t}}} }} = \frac{1}{{{\text{k}}_{2} {\text{Q}}_{{\text{e}}}^{2} }} + \frac{{\text{t}}}{{{\text{Q}}_{{\text{e}}} }} $$

where *k*_*i*_, *k*_*1*_, and *k*_*2*_ are intraparticle diffusion rate constant (mg g^−1^ min^−1/2^), pseudo-first-order rate constant (min^−1^), and pseudo-second-order rate constant (g mg^−1^ min^−1^) of adsorption, respectively. *Q*_*t*_ and *Q*_*e*_ are respectively the adsorption capacity (mg g^−1^) at time t (min) and equilibrium time. *C* (mg g^−1^) represents a constant for the intra-particle diffusion model^51–53^. In order to investigate the kinetic parameters, four different times close the optimum time was selected for each analyte, and the adsorption of these different times was investigated under optimal conditions of other effective parameters. To describe the adsorption mechanism, the desired isotherm was determined according to the correlation coefficient of the linear model of the different kinetic equations. Table 3 shows the kinetic parameters obtained from these models with various pollutants by ZF-NPs. As seen, a good fit is represented by the pseudo-second-order model for the experimental kinetic data in terms of the correlation coefficient (R) for each kinetic model. At the same time, there is consistency between the equilibrium adsorption capacities determined in terms of the pseudo-second-order model and the experimental data for each pollutant. Thus, using organic dyes and heavy metals ions on ZF-NPs is fitted perfectly with the pseudo-second-order model compared to the other models. In rate-determining step is assumed to be chemisorption in the pseudo-second-order model, including valence forces by exchanging or sharing sorbate and sorbent electrons. Moreover, the adsorption capacity is proportionate to the number of active sites engaged over the adsorbent surface. Hence, it is indicated that the pollutants adsorption on ZF-NPs is mostly the chemical reactive adsorption.

**Table 3:** Parameters of kinetics model for adsorption of AO, MB, and Cd (II) on ZF-NPS.

Pollutants	Q_exp_ (mg g^−1^)	Pseudo-first-order			Pseudo-second-order			Intraparticle diffusion	
		k_1_ × 10^2^ (min^−1^)	Qe (mg g^−1^)	R	k_2_ × 10^2^ (g g^−1^ min^−1^)	Q_e_ (mg g^−1^)	R	K_i_ (mg g^−1^ min^−1/2^)	R
AO	110.28	2.58	103.14	0.8881	0.28	114.51	0.9948	6.3619	0.9203
MB	128.04	0.96	24.51	0.8897	2.614	132.49	0.9998	3.4094	0.6812
Cd (II)	105.45	3.82	21.68	0.5943	1.65	109.07	0.9985	5.6927	0.5903

### The point of zero charge of ZF-NPS

**Figure 4:**
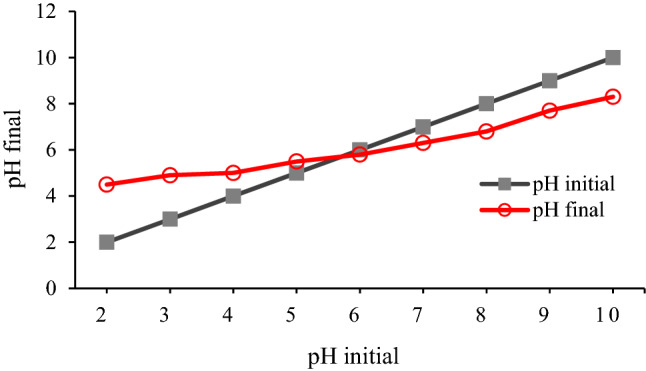
The pH_pzc_ of ZF-NPS.

PZC point is one of the important properties for determining the charge of the adsorbent surface. This parameter denotes the pH value of the media around the adsorbent, at which the net positive surface charges are in equilibrium with the net negative surface charges, resulting in a zero-surface charge (actually, the pH at which the net surface charge is equal to zero is called PZC). In order to determine the pHpzc value, 50 mL of 0.01 M NaCl was transferred into several Erlenmeyer flasks, and the pH of these solutions was adjusted in the range of 2–10 by 0.1 M HCl and 0.1 M NaOH. Then, 0.1 g of adsorbent was added to each flask and stirred for 2 h on a shaker. After 3 h, the pH of each solution was measured with a pH meter, and the diagram of final pH versus the initial pH was plotted (Fig. 4). The intersection of these two curves is called pHpzc, which is equal to 5.8 for ZF-NPS.

### Optimizing the effective parameters of the process

These experiments and designing the experiment are performed to achieve the optimum point with the highest removal percentage. After obtaining the response of the 30 designed experiments, the Design-Expert software analyzed the responses. In addition to the calculations performed, a few new experiments were proposed as the points at which a more acceptable response is likely to be obtained. These suggested points of the software can be seen in Table 4. Under optimum conditions, the removal efficiency of 95.81% for AO, 98.62% for MB, and 92.20% for Cd (II) were achieved applying ZF-NPS (the average values considered as the final results).

**Table 4:** Optimum conditions of removal of AO, MB, and Cd (II).

Run	Variables				%Removal			
	A (g)	B (mg L^−1^)	C	D (min)	AO	MB		Cd (II)
1	0.25	15	6	40	95.72	98.29		91.56
2	0.25	15	6	40	96.08	98.45		92.69
3	0.25	15	6	40	95.63	99.13		92.37

### Reusability of adsorbent

**Figure 5:**
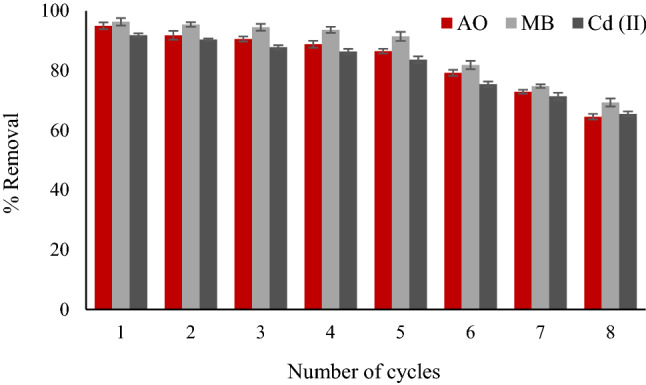
Desorption performance of ZF-NPS in seven regeneration cycles (Removal conditions: adsorbent amount 0.25 g, pH = 6, sonication time 15 min, and concentration 15 mg L^−1^).

Adsorbent reusability is an important parameter that allows the adsorbent to be reused over and over again. This property of adsorbent saves a lot of time and money, and this is, in turn, a great ability, so this property was verified. For this purpose, the experiments were run according to the described procedure in “Batch experiments” section. Upon completion of the reaction, the adsorbent particles were separated from the reaction mixture by an external magnetic field. The particles were washed several times with distilled water, dried in an oven at 60 °C. After complete drying of the particles, they were reused, and the adsorption and desorption steps were repeated up to 8 runs. In all stages, the final concentration of dyes was measured by UV–Vis spectrophotometer, and the final concentration of heavy metals was measured by atomic absorption spectroscopy (AAS). The reusability results are shown in Fig. 5. It was found that this adsorbent can be reused up to 5 times without any significant changes in its efficiency.

### Real sample analysis

In order to investigate the removal of AO, MB, and Cd (II) by ZF-NPS, various samples such as tap water, university of Tehran wastewater, and channel water (untreated water) were used. Preliminary experiments displayed that no measurable amounts of heavy metals and dyes were detected in these samples. To evaluate the applicability of the proposed method, the samples were filtered and contaminated with some of each analyte. Then, the amounts of pollutants removed under optimum conditions mentioned in “Batch experiments” section was measured. The obtained results in Table 5 show that the adsorbent has high selectivity for removing AO, MB, and Cd (II) from ambient water samples.

**Table 5:** Removal of AO, MB, and Cd (II) in real samples.

Real samples	%R_AO_ ± RSD	%R_MB_ ± RSD	%R_Cd(II)_ ± RSD
Tap water	93.58 ± 3.41	97.49 ± 1.96	92.27 ± 2.38
University of tehran wastewater	91.20 ± 3.08	93.09 ± 3.18	85.93 ± 1.89
Channel water (untreated water)	89.75 ± 3.63	92.04 ± 3.40	83.19 ± 2.74

### Interference studies

Since there are many ions in environmental wastewater samples, and the presence of these ions may have a negative or positive effect on the removal of dye and metal, in this section, the presence effect of these species was probed. In this regard, different concentrations of ions were added to the test solution, and the removal rate under optimum conditions was measured according to “Batch experiments” section. A species that results in a ± 5% error in the analyte adsorption signal is known as an interfering ion. The obtained results are given in Table 6. The results show that if the concentration of interfering metal ions is 100 times greater than the concentration of analyte, a significant reduction in the removal of AO, MB, and Cd (II) will occur.

**Table 6:** Effect of interfering ions on the removal of AO, MB, and Cd (II).

Interference	Tolerance (mg L^−1^)	%RAO	%RMB	%RCd(II)
Ni (II)	0	95.81	98.62	92.20
	50	92.76	97.69	90.96
	100	87.60	91.39	86.83
Fe (III)	0	95.81	98.62	92.20
	50	94.55	95.02	92.39
	100	88.16	89.86	85.16
Zn (II)	0	95.81	98.62	92.20
	50	95.04	97.42	91.97
	100	88.68	89.63	84.89
Al (III)	0	95.81	98.62	92.20
	50	95.04	97.76	92.87
	100	88.40	92.07	85.61
Co (II)	0	95.81	98.62	92.20
	50	96.32	99.13	91.45
	100	89.68	90.57	85.08

## Conclusion

Due to the colossal importance of water resources in human life and other organisms, preventing aquatic environment pollution is of concern. Dyes and heavy metals are some of the most harmful pollutants in water sources. The selected method for removing these pollutants should be time and cost savings and cause the least environmental damage. In this study, an adsorbent with high efficiency in removing AO, MB, and Cd (II) by using low-cost, available, and environmentally friendly materials is reported. To achieve time and cost savings and the least error response, the CCD-based RSM method was chosen for the optimization. Quadratic models for removal of dyes and heavy metal were statistically compared with values of *p* < 0.0001 and R^2^ ˃ 0.99, and the results showed that models have reasonable accuracy. Optimal conditions were obtained at sonication time of 15 min, adsorbent amount of 0.25 g, pH = 6, and concentration of 15 mg L^−1^. The ZF-NPS adsorbent can remove 95.81% of AO, 98.62% of MB, and 92.20% of Cd (II) from an aqueous solution under optimum conditions. These values indicate the high efficiency of the adsorbent in the removal of these pollutants. The adsorption kinetics was well described by the pseudo-second-order model. Equilibrium data fitted well with the Langmuir model. The maximum adsorption capacity reached 201.29 mg g^−1^, 256.76 mg g^−1^ and 152.48 mg g^−1^ for AO, MB, and Cd (II), respectively. In order to cost-saving in preparing this adsorbent, its reusability was investigated. It was found that after five repetitive runs, the maximum removal percentage achieved for all three analytes was more than 90%. The use of ZF-NPS on real samples revealed that ZF-NPS could remove AO, MB, and Cd (II) in the range of 83.19–97.49%. Moreover, interference studies showed that if the concentration of interfering metal ions is 100 times greater than the concentration of analyte, a significant reduction in the removal of AO, MB, and Cd (II) will occur. Therefore, according to the obtained results, ZF-NPS is a highly efficient, low cost, low dosage, high durable, and promising adsorbent for AO, MB, and Cd (II) removal.

## Supplementary Information

Supplementary Information.

## Data Availability

The authors declare that [the/all other] data supporting the findings of this study are available within the paper [and its supplementary information files].
