# Supplementary material for: Experimental modelling studies on the removal of dyes and heavy metal ions using ZnFe2O4 nanoparticles
Source: Sci Rep. 2022 Apr 9;12:5987. doi: 10.1038/s41598-022-10036-y (PMC8994778; doi:10.1038/s41598-022-10036-y)
Supplement: Supplementary file 1 — Supplementary Information. [file 41598_2022_10036_MOESM1_ESM.docx]

**Supplementary information files**

**Table S1.** Experimental results and the predicted values of percentage removal.

**Table S2.**  Analysis of variance (ANOVA) of remove AO and MB.

**Table S3.**  Analysis of variance (ANOVA) of remove Cd (II).

**Fig. S1.** XPS spectra of ZF-NPS: a) survey, b) O 1s, c) Zn 2p, and d) Fe 2p.

**Fig. S2.** Plot of predicted versus actual for removal of a) AO, b) MB c) Cd (II).

**Table S1.** Experimental results and the predicted values of percentage removal.

|  | %R_AO_ | | | %R_MB_ | | | %R_Cd (II)_ | |
| --- | --- | --- | --- | --- | --- | --- | --- | --- |
| Run | Experimental | Predicted | Experimental | | Predicted | Experimental | | Predicted |
| 1 | 84.36 | 82.48 | 95.09 | | 92.70 | 82.61 | | 82.63 |
| 2 | 54.76 | 53.73 | 50.47 | | 50.22 | 55.64 | | 57.96 |
| 3 | 33.05 | 30.79 | 25.23 | | 23.64 | 43.18 | | 41.53 |
| 4 | 74.13 | 73.44 | 67.68 | | 67.11 | 80.41 | | 77.96 |
| 5 | 36.41 | 37.70 | 33.19 | | 36.21 | 39.36 | | 40.89 |
| 6 | 33.95 | 34.72 | 39.50 | | 38.82 | 37.57 | | 39.26 |
| 7 | 18.37 | 18.48 | 19.94 | | 18.54 | 25.61 | | 26.53 |
| 8 | 82.23 | 82.48 | 92.42 | | 92.70 | 82.05 | | 82.63 |
| 9 | 81.7 | 82.48 | 93.11 | | 92.70 | 84.39 | | 82.63 |
| 10 | 61.45 | 58.66 | 65.41 | | 65.80 | 66.29 | | 64.25 |
| 11 | 52.09 | 52.15 | 48.75 | | 50.86 | 52.50 | | 54.17 |
| 12 | 45.37 | 43.21 | 55.76 | | 56.27 | 48.07 | | 46.06 |
| 13 | 53.86 | 54.38 | 61.31 | | 60.42 | 56.40 | | 57.21 |
| 14 | 52.25 | 53.85 | 47.69 | | 50.19 | 49.61 | | 51.37 |
| 15 | 69.57 | 70.65 | 74.08 | | 72.01 | 69.59 | | 69.67 |
| 16 | 93.61 | 94.07 | 95.20 | | 97.82 | 90.17 | | 91.73 |
| 17 | 34.37 | 36.29 | 33.45 | | 35.15 | 43.58 | | 44.47 |
| 18 | 73.53 | 75.13 | 71.09 | | 72.72 | 72.72 | | 72.65 |
| 19 | 50.72 | 52.33 | 58.38 | | 59.84 | 60.87 | | 61.03 |
| 20 | 82.46 | 82.48 | 91.42 | | 92.70 | 81.16 | | 82.63 |
| 21 | 46.92 | 46.38 | 43.85 | | 43.29 | 47.23 | | 44.29 |
| 22 | 81.03 | 82.48 | 92.28 | | 92.70 | 83.59 | | 82.63 |
| 23 | 78.34 | 77.55 | 85.14 | | 82.46 | 73.86 | | 71.77 |
| 24 | 83.11 | 82.48 | 91.89 | | 92.70 | 81.97 | | 82.63 |
| 25 | 74.35 | 76.40 | 87.33 | | 86.54 | 77.81 | | 79.20 |
| 26 | 34.83 | 35.15 | 31.71 | | 33.45 | 39.48 | | 39.70 |
| 27 | 87.81 | 85.40 | 88.42 | | 86.81 | 81.91 | | 80.75 |
| 28 | 60.95 | 59.93 | 59.19 | | 57.91 | 58.34 | | 59.06 |
| 29 | 72.42 | 72.87 | 71.65 | | 70.90 | 72.50 | | 73.22 |
| 30 | 23.63 | 23.48 | 22.98 | | 20.43 | 34.49 | | 32.44 |

**Table S2.**  Analysis of variance (ANOVA) of remove AO and MB.

| Source | DF | AO | | | | | | | | |  | MB | | | | | |
| --- | --- | --- | --- | --- | --- | --- | --- | --- | --- | --- | --- | --- | --- | --- | --- | --- | --- |
|  |  | Sum of squares | | | Mean square | | | F-value | P-value | |  | Sum of squares | | Mean square | F-value | P-value | |
| Model | 14 | 13024.84 | | | 930.35 | | | 261.05 | < 0.0001 | |  | 17508.78 | | 1250.63 | 246.68 | < 0.0001 | |
| A- Adsorbent amount | 1 | 2728.53 | | | 2728.53 | | | 765.60 | < 0.0001 | |  | 2834.68 | | 2834.68 | 559.12 | < 0.0001 | |
| B- Analyte concentration | 1 | 2283.84 | | | 2283.84 | | | 640.82 | < 0.0001 | |  | 2841.64 | | 2841.64 | 560.49 | < 0.0001 | |
| C- pH of the solution | 1 | 1856.10 | | | 1856.10 | | | 520.80 | < 0.0001 | |  | 3087.43 | | 3087.43 | 608.97 | < 0.0001 | |
| D- Sonication time | 1 | 1768.51 | | | 1768.51 | | | 496.23 | < 0.0001 | |  | 1028.48 | | 1028.48 | 202.86 | < 0.0001 | |
| AB | 1 | 19.45 | | | 19.45 | | | 5.46 | 0.0338 | |  | 25.93 | | 25.93 | 5.12 | 0.0390 | |
| AC | 1 | 0.19 | | | 0.19 | | | 0.054 | 0.8189 | |  | 15.50 | | 15.50 | 3.06 | 0.1008 | |
| AD | 1 | 0.42 | | | 0.42 | | | 0.12 | 0.7373 | |  | 0.80 | | 0.80 | 0.16 | 0.6974 | |
| BC | 1 | 0.040 | | | 0.040 | | | 0.011 | 0.9170 | |  | 19.51 | | 19.51 | 3.85 | 0.0686 | |
| BD | 1 | 0.66 | | | 0.66 | | | 0.19 | 0.6721 | |  | 0.64 | | 0.64 | 0.13 | 0.7281 | |
| CD | 1 | 6.08 | | | 6.08 | | | 1.70 | 0.2113 | |  | 12.51 | | 12.51 | 2.47 | 0.1370 | |
| A^2^ | 1 | 1580.63 | | | 1580.63 | | | 443.51 | < 0.0001 | |  | 3840.29 | | 3840.29 | 757.47 | < 0.0001 | |
| B^2^ | 1 | 471.82 | | | 471.82 | | | 132.39 | < 0.0001 | |  | 1310.65 | | 1310.65 | 258.52 | < 0.0001 | |
| C^2^ | 1 | 2940.35 | | | 2940.35 | | | 825.03 | < 0.0001 | |  | 4215.79 | | 4215.79 | 831.53 | < 0.0001 | |
| D^2^ | 1 | 837.27 | | | 837.27 | | | 234.93 | < 0.0001 | |  | 933.50 | | 933.50 | 184.13 | < 0.0001 | |
| Residual | 15 | 53.46 | | | 3.56 | | |  |  | |  | 76.05 | | 5.07 |  |  | |
| Lack of Fit | 10 | 46.75 | | | 4.68 | | | 3.49 | 0.0903 | |  | 67.62 | | 6.76 | 4.01 | 0.0693 | |
| Pure Error | 5 | 6.71 | | | 1.34 | | |  |  | |  | 8.43 | | 1.69 |  |  | |
| Cor Total | 29 | 13078.30 | | |  | | |  |  | |  | 17584.83 | |  |  |  | |
| Model Summary Statistics | | | | | | | | | | | | | | | | |  |
|  | | | AO | | | | | | |  | | | MB | | | |  |
| Precision | | | R^2^ | R^2^-Adj | | | R^2^-Pred | | |  | | | R^2^ | R^2^-Adj | R^2^-Pred | |  |
|  |  |  | 0.9959 | 0.9921 | | 0.9787 | | | |  | | | 0.9957 | 0.9916 | 0.9772 | |  |

**Table S3.**  Analysis of variance (ANOVA) of remove Cd (II).

| Source | DF | Cd (II) | | | | | | | | |  |  |
| --- | --- | --- | --- | --- | --- | --- | --- | --- | --- | --- | --- | --- |
|  |  | Sum of squares | | | Mean square | | | F-value | P-value | |  |  |
| Model | 14 | 9842.05 | | | 703.00 | | | 158.18 | < 0.0001 | |  |  |
| A- Adsorbent amount | 1 | 1990.90 | | | 1990.90 | | | 447.98 | < 0.0001 | |  |  |
| B- Analyte concentration | 1 | 1994.18 | | | 1994.18 | | | 448.71 | < 0.0001 | |  |  |
| C- pH of the solution | 1 | 1518.29 | | | 1518.29 | | | 341.63 | < 0.0001 | |  |  |
| D- Sonication time | 1 | 991.38 | | | 991.38 | | | 223.07 | < 0.0001 | |  |  |
| AB | 1 | 0.047 | | | 0.047 | | | 0.011 | 0.9192 | |  |  |
| AC | 1 | 12.87 | | | 12.87 | | | 2.90 | 0.1094 | |  |  |
| AD | 1 | 15.15 | | | 15.15 | | | 3.41 | 0.0847 | |  |  |
| BC | 1 | 14.65 | | | 14.65 | | | 3.30 | 0.0895 | |  |  |
| BD | 1 | 12.09 | | | 12.09 | | | 2.72 | 0.1198 | |  |  |
| CD | 1 | 1.12 | | | 1.12 | | | 0.25 | 0.6232 | |  |  |
| A^2^ | 1 | 897.45 | | | 897.45 | | | 201.94 | < 0.0001 | |  |  |
| B^2^ | 1 | 692.96 | | | 692.96 | | | 155.92 | < 0.0001 | |  |  |
| C^2^ | 1 | 2015.13 | | | 2015.13 | | | 453.43 | < 0.0001 | |  |  |
| D^2^ | 1 | 963.74 | | | 963.74 | | | 216.85 | < 0.0001 | |  |  |
| Residual | 15 | 66.66 | | | 4.44 | | |  |  | |  |  |
| Lack of Fit | 10 | 59.71 | | | 5.97 | | | 4.29 | 0.0607 | |  |  |
| Pure Error | 5 | 6.95 | | | 1.39 | | |  |  | |  |  |
| Cor Total | 29 | 9908.71 | | |  | | |  |  | |  |  |
| Precision | | | R^2^ | R^2^-Adj | | | R^2^-Pred | | |  | | |
|  |  |  | 0.9933 | 0.9870 | | 0.9643 | | | |  | | |

**
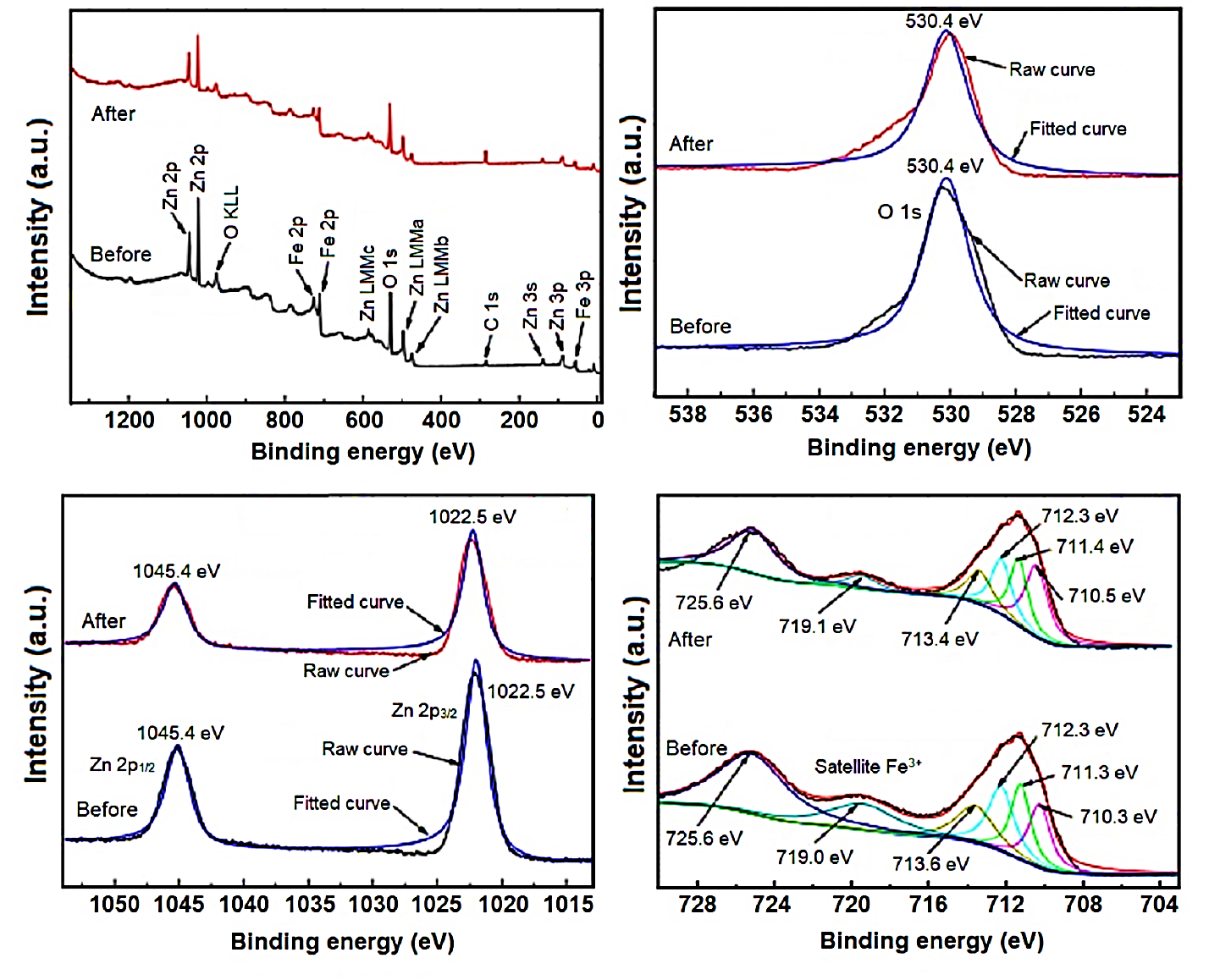
**

c)

d)

a)

b)

**Fig. S1.** XPS spectra of ZF-NPS: a) survey, b) O 1s, c) Zn 2p, and d) Fe 2p.

|   b) | |   a) |
| --- | --- | --- |
|  |   c) | |
| **Fig. S2.** Plot of predicted versus actual for removal of a) AO, b) MB c) Cd (II). | | |
